# New dimension in therapeutic targeting of BCL-2 family proteins

**DOI:** 10.18632/oncotarget.3868

**Published:** 2015-04-19

**Authors:** Samaher Besbes, Massoud Mirshahi, Marc Pocard, Christian Billard

**Affiliations:** ^1^ INSERM U 965, Hôpital Lariboisière, Paris, France; ^2^ Université Paris Diderot, UMR S965, Paris, France

**Keywords:** anticancer therapy, apoptosis, targeting BCL-2 family proteins, BH3 mimetics, prosurvival protein antagonists

## Abstract

Proteins of the BCL-2 family control the mitochondrial pathway of apoptosis. Targeting these proteins proves to be an attractive strategy for anticancer therapy. The biological context is based on the fact that BH3-only members of the family are specific antagonists of prosurvival members. This prompted the identification of “BH3 mimetic” compounds. These small peptides or organic molecules indeed mimic the BH3 domain of BH3-only proteins: by selectively binding and antagonizing prosurvival proteins, they can induce apoptosis in malignant cells. Some small-molecule inhibitors of prosurvival proteins have already entered clinical trials in cancer patients and two of them have shown significant therapeutic effects. The latest developments in the field of targeting BCL-2 family proteins highlight several new antagonists of prosurvival proteins as well as direct activators of proapoptotic proteins. These compounds open up novel prospects for the development of BH3 mimetic anticancer drugs.

## INTRODUCTION

Apoptosis is a cell death program intended for regulating cell number during development and tissue homeostasis as well as eliminating damaged/dangerous cells [1]. Evasion of apoptosis is typical of many cancers and a frequent cause of therapeutic resistance [2, 3]. This evasion is mostly due to impairment in the mitochondrial pathway of apoptosis. Proteins of the BCL-2 family strictly control this pathway by inducing the mitochondrial outer membrane permeabilization (MOMP). The latter indeed allows the release of apoptogenic factors responsible for the activation of caspases that are the executioners of the cellular demolition program [1, 2].

The BCL-2 family proteins are characterized by their domains of homology to BCL-2 (BH1 to 4) and their functional activities. They comprise the prosurvival members (BCL-2, BCL-X_L_, BCL-W, MCL-1 and A1) and the proapoptotic members. The latter include the MOMP effectors (mainly BAX and BAK) and the BH3-only proteins, so-called because they have only the BH3 domain (e.g., BIM, BID, PUMA, BAD, NOXA). The activity of the prosurvival proteins is to bind to proapoptotic molecules, notably the MOMP effectors BAX and BAK which are thus sequestered in an inactive form. All prosurvivals can bind BAX whereas only BCL-X_L_ and MCL-1 bind to BAK. This antiapoptotic activity is antagonized by the BH3-only members: by inserting their helical BH3 region into the hydrophobic groove of the prosurvival proteins, they provoke the release of the sequestered BAX and BAK that are thus activated. These interactions are selective: for example NOXA binds only to MCL-1 and A1, BAD binds only to BCL-2, BCL-X_L_ and BCL-W, whereas BIM, BID and PUMA can bind to all prosurvival proteins [1, 2]. Certain BH3-only proteins (such as BIM, BID and probably PUMA) can directly activate the proapoptotic proteins BAX and BAK.

Hence, the BCL-2 family proteins govern the MOMP by their specific interactions, and thus the fate of cells to either live or die. Targeting these proteins therefore appeared as an attractive apoptosis-based strategy to assist anticancer therapy [1, 2]. A particular attention has been focused on the development of agents capable of inhibiting the activity of prosurvival BCL-2 family members that are overexpressed in various malignancies [1, 2, 4]. This has led to the characterization of small molecules mimicking the BH3 domain of BH3-only proteins, likely to selectively bind to and antagonize prosurvival family members and thus induce apoptosis. These “BH3 mimetic” compounds are short peptides modeled on BH3 domains and small organic molecules. The latter are identified by screening libraries of either natural products or computer-designed molecules for binding to BCL-2 proteins [5-16]. Two major criteria were proposed by Lessene *et al* to define an authentic BH3 mimetic: high affinity binding to the targets (in the nM range) and induction of BAX/BAK-dependent apoptosis [5]. Some compounds have shown significant therapeutic effects in cancer patients.

The preclinical and clinical properties of the small molecules inhibiting prosurvival BCL-2 family proteins have been extensively reviewed [5-16]. Two of the most recent reviews have described the biological context to targeting these proteins and advances in therapeutic approaches with BH3 mimetics. In the one, Anderson *et al* have focused on the four agents that are in clinical evaluation, discussed the data in detail and pinpointed questions yet to be resolved for using these agents as part of combination therapy [15]. In the other, Roy *et al* have presented a comprehensive review of compounds that target the BCL-2 family-driven pathway [16]. The present article updates the small molecules targeting proteins of the BCL-2 family with the discovery of not only highly potent antagonists of prosurvival members but also direct activators of the MOMP effectors BAX and BAK and a dual prosurvival inhibitor/proapoptotic activator. These data bring a new dimension to the therapeutic targeting of BCL-2 family proteins.

## INHIBITORS OF PROSURVIVAL BCL-2 PROTEINS

### Small organic molecules

#### Obatoclax

This synthetic indol bipyrrole molecule derived from the natural product prodigiosin is capable of binding to all prosurvival BCL-2 family proteins with low affinity (in the μM range) and inducing apoptosis in tumor cells *in vitro* [17]. This putative pan-BH3 mimetic (or BIM-like BH3 mimetic) was the first to enter clinical trials but has shown only modest therapeutic effects [15, 18]. It is now known that obatoclax does not meet the two main criteria defining an authentic BH3 mimetic and that its proapoptotic activities result from off-target mechanisms [19, 20].

#### Gossypol family

Gossypol, a natural polyphenol, and its synthetic isomer AT-101 [21, 22] are also putative pan-BH3 mimetics: they do not fully meet the criteria for a *bona fide* BH3 mimetic and induce apoptosis via multiple mechanisms [19, 20, 23]. Like obatoclax, they showed limited anticancer activity in clinical trials [15]. Several gossypol and AT-101 derivatives such as sabutoclax (BI-97C1) and BI-97D6 were characterized in preclinical studies as exhibiting higher binding affinities (in the sub-μM range) and triggering predominantly BAX/BAK-dependent apoptosis; both sabutoclax and BI-97D6 show *in vivo* antitumor effects in animal models [24, 25]. Interestingly, sabutoclax has turned out to be a pan-BCL-2 inhibitor in some but not all cellular systems, displaying its best activity in inhibiting MCL-1 [26].

TW-37, a rationally designed benzoylsulphonyl analog of gossypol [22, 27], was also known to operate only in part as a pan-BH3 mimetic: it binds to BCL-2, BCL-X_L_ and MCL-1 with moderate affinity (sub-μM), induces apoptosis depending partially on BAX/BAK activation and shows several off-target effects. However, a recent careful analysis has demonstrated that TW-37 (i) induces several typical features of mitochondrial apoptosis in MCL-1-dependent cells [but not BCL-2 or BCL-X_L_-dependent cells] and (ii) exhibits all the hallmarks of a NOXA-like BH3 mimetic antagonizing selectively MCL-1, although only at high concentrations [26]. This study suggested that derivatives of TW-37 with higher affinity for MCL-1 might be developed [26].

#### ABT-737 and navitoclax

The fragment-screening approach based on structure/activity relationship (SAR) by nuclear magnetic resonance (NMR) - initially described by Fesik and colleagues [28] - led to the discovery of ABT-737, a molecule with an acylsulfonamide moiety [29]. Its orally-bioavailable derivative ABT-263 (now navitoclax) was designed for clinical use [30]. Both molecules are authentic BH3 mimetics targeting BCL-2, BCL-X_L_ and BCL-W but not MCL-1 or A1 (as the BH3-only protein BAD, so they are referred to as BAD-like BH3 mimetics). They were extensively characterized in preclinical studies [5, 16, 23]. The therapeutic activity of navitoclax in patients with hematologic malignancies (particularly chronic lymphocytic leukemia) and some solid cancers is now well established [15, 16].

#### ABT-199

Thrombocytopenia (i.e., an abnormal decrease in number of platelets in the blood) is a dose-limiting effect of navitoclax due to the inhibition of BCL-X_L_ (a survival factor for platelets). To circumvent this side effect, navitoclax derivatives that bind specifically to BCL-2 (without significant binding to BCL-X_L_ or BCL-W) were designed, leading to ABT-199. This true BH3 mimetic is the first highly selective inhibitor of BCL-2 [31]. Initial clinical studies of ABT-199 in chronic lymphocytic leukemia (CLL) and non-Hodgkin's lymphoma have already shown impressive antitumor efficacy, with higher response rates than navitoclax and without thrombocytopenia [15, 16, 31]. The latest results from the trial in refractory or relapsed CLL patients have confirmed the effectiveness of ABT-199: 23% of the 78 patients recruited for 2 years had complete response (with no evidence of cancer in some patients), 54 % achieved a partial response and overall, 59% of the patients have survived for 2 years without leukemia progression; in addition, the complete response rates were similar in high-risk patients including those having p53 deficiency due to the deletion in chromosome 17 [32]. Moreover, several promising clinical data have been lastly reported: notably in phase I/II trials of ABT-199 in acute myeloid leukemia, and of its combination with bendamustine, rituximab or obinituzumab in non-Hodgkin's lymphoma and CLL [33-36]. Phase III studies of ABT-199 (now venetoclax) are ongoing.

#### Other ABT-737 derivatives

Isosteric analogs of ABT-737 in which the acylsulfonamide moiety is replaced by a quinazoline group were synthesized: these quinazoline derivatives have reduced capacities to bind BCL-W while keeping their high-affinity binding to BCL-2 and BCL-X_L_ [37]. The same characteristics were observed with other acylsulfonamide derivatives, as previously reviewed [16].

#### BM-957 and BM-1197

BM-957 is another ABT-737 derivative (a diphenyl-pyrrole-carboxamide scaffold linked to the nitrobenzene sulfonamide half of ABT-737): it also binds to BCL-2 and BCL-X_L_ with sub-nM affinity, induces caspase 3-dependent apoptosis in tumor cell lines and causes tumor regression in xenograft models [38]. In order to optimize their compound BM-957, Wang and colleagues performed modifications of the pyrrole-carboxylic acid core structure leading to the new small molecule BM-1197 [39]. This compound binds to BCL-2 and BCL-X_L_ with sub-nM affinity, inhibits their antiapoptotic activities and induces BAX/BAK-dependent apoptosis in small cell lung cancer cell lines. In addition, BM-1197 shows improved solubility and pharmacokinetic properties and elicits complete and long-term tumor regression in two different small cell lung cancer xenograft models [39]. Consequently, BM-1197 proves to be a new BAD-like BH3 mimetic.

#### WEHI-539 and A-1155463

High-throughput screening of small molecules targeting BCL-X_L_ and structure-guided design led to the discovery of WEHI-539, a molecule with a benzothiazole hydrazone scaffold. This compound was the first highly selective antagonist of BCL-X_L_ (through high affinity binding) over BCL-2 and BCL-W. Careful analysis of its mechanism of action has shown that WEHI-539 induces BAK-mediated apoptosis [40]. NMR fragment screening and structure-based design further led to the characterization of A-1155463, a more potent BCL-X_L_ inhibitor than WEHI-539 while possessing none of its pharmaceutical liabilities. Importantly, this compound acts through an on-target mechanism and shows *in vivo* antitumor activity in mice [41]. The new small molecule A-155463 constitutes an actual BH3 mimetic specific for BCL-X_L_.

#### Arylsulfonamide derivatives

Hybridization of the arylsulfonamide portion of ABT-737 with an MCL-1 inhibitor resulted in a dual BCL-X_L_/MCL-1 ligand with moderate (sub-μM) affinity [42]. A substituted arylsulfonamide has shown selectivity for MCL-1 (albeit with sub-μM binding affinity): this molecule induces caspase-9 activation and apoptosis [43]. Interestingly, an approach similar to that used for the ABT series has recently allowed the design of a biphenyl arylsulfonamide molecule also targeting MCL-1 with an apparent affinity of 30 nM; despite it is not known whether this compound can induce apoptosis, it might serve as a promising lead for optimized effects [44].

#### MCL-1 inhibitor molecule-1 (MIM-1)

High-throughput screening of small molecules displacing a stapled peptide derived from the BH3 domain of MCL-1 has yielded a substituted thiazol molecule called MIM-1 (for MCL-1 inhibitor molecule-1); this small molecule specifically engages the binding groove of MCL-1 and induces BAK-dependent apoptosis in MCL-1-dependent cells [45]. However, the binding affinity for MCL-1 is modest (4.8 μM), high concentrations of MIM-1 are required for apoptosis, and this activity depends on the cell type [26].

#### Small molecule MCL-1 inhibitor

Using their NMR-based fragment screen approach (having led to ABT-737), Fesik and colleagues recently identified two chemically distinct compounds that bind to different sites of MCL-1; they then merged these compounds together to produce molecules binding MCL-1 selectively over BCL-2 and BCL-X_L_ [46]. After optimization, they obtained a lead compound [indole-2-carboxylic acid] which binds and inhibits MCL-1 (with an affinity of 55 nM) and induces apoptosis. Crystal structure of the complex validates the strategy and provides detailed information to design more potent inhibitors [46].

#### A-1210477

A series of specific MCL-1 inhibitors exhibiting sub-nM affinity were obtained by high throughput screening and structure-guided design [47]. The compounds are indole-2-carboxylic acids like the small molecule MCL-1 inhibitor described by Fesik and colleagues [46], but have a 100-fold higher binding affinity. Leverson *et al* have further characterized the most potent of the compounds, A-1210477: it binds selectively to MCL-1 with an affinity of 0.45 nM, disrupts BIM/MCL-1 complexes in living cells, induces the hallmarks of mitochondrial apoptosis in MCL-1-dependent cancer cells and synergizes with navitoclax to induce apoptosis in various malignant cell lines [48]. Data also demonstrate that the compound acts through an on-target mechanism. Therefore, A-1210477 appears as the first BH3 mimetic targeting MCL-1.

#### Mcl-1 inhibitor-9

A high-throughput screening approach culminated in the characterization of a piperazine-substituted hydroxyquinoline that inhibits selectively MCL-1. This molecule called Mcl-1 inhibitor-9 was proposed to function as a BH3 mimetic in inducing mitochondrial apoptosis in cell lines [49] although the binding affinity and mechanism of action have not been evaluated.

#### Sesquiterpenoid derivatives

Screening a library of natural sesquiterpenoids for disrupting the MCL-1/BIM BH3 complex led to the identification of hexahydronaphtalene and the synthesis of substituted derivatives. The most potent MCL-1 inhibitor can engage the binding groove of MCL-1 in computational docking experiments, and it shows antagonizing activity in cancer cells [50]. This possible new class of MCL-1 inhibitor yet needs further development and biological characterization.

#### S1 family

The small molecule S1 (an oxo-phenalene-dicarbonitrile) binds to both BCL-2 and MCL-1 albeit with moderate affinity (in the sub-μM range), triggers BAX/BAK-dependent apoptosis, and shows antitumor activity in an animal model [51]. Although initially proposed as a pan-BH3 mimetic, S1 was reportedly found to act via multiple and off-target mechanisms [20, 52, 53]. A substituted derivative of S1 was designed and characterized as showing a higher affinity for binding MCL-1 (10 nM) and BCL-2 (20 nM) and a better apoptotic activity in tumor cell lines than the parental S1 [54]. Unless the demonstration that this activity is BAX/BAK-dependent, S1-derivative should be considered as a putative pan-BH3 mimetic. Other S1-derivatives were described as novel MCL-1 inhibitors [55, 56].

#### Acylsulfonamide molecules

A fragment-based NMR method (other than the Fesik's approach) has allowed the generation of a series of acylsulfonamides (different from the ABT series). The small molecules exhibit high affinity (nM) for both BCL-X_L_ and MCL-1 [57]. However, their biological validation has not been provided.

#### Compound JY-1-106

SAR analysis of oligoamide foldamers led to the design of JY-1-106, a substituted trisarylamide having the ability to induce apoptosis *in vitro* and to repress tumor growth in an animal model. Computational analyses indicate that JY-1-106 engages the BH3-binding grooves of both BCL-X_L_ and MCL-1, suggesting that it may be a pan-BH3 mimetic [58]. Whether JY-1-106 meets the two criteria that define an authentic BH3 mimetic yet remains to be stated.

#### Other new small molecules

A number of small organic molecules designed to mimic BH3 peptides have just been reported such as a series of molecules containing a benzoylurea scaffold which selectively bind to BCL-X_L_ albeit with μM affinity [59]. Is is also the case for several MCL-1 inhibitors including marinopyrrole derivatives, a single diastereomer of a macrolactam core and compounds with a 3-phenylthiophene-2-sulfonamide core moiety or with a phenyl-piperazine-triazine scaffold [60-63]. Interestingly, a small molecule targeting the prosurvival BCL-2 family protein A1 has been identified for the first time [64]. Although these compounds require further characterization and improvements, they may represent starting points for developing new classes of inhibitors.

### BH3 domain-derived peptides

Peptides hardly penetrate into cells and are prone to rapid proteolytic degradation. Efforts focused to modify peptides in order to increase the cell penetration and stability and also to reproduce the helicity of BH3 domains needed for high affinity binding.

#### α/β foldamers

BIM and PUMA BH3 peptides were modified by incorporating non-natural amino acids: this resulted in α/β foldamer-peptides capable of binding to BCL-X_L_ [65] and to both BCL-X_L_ and MCL-1 with high affinity [66], as previously discussed [16]. It is furthermore noteworthy that peptides with non-canonical BH3 domain sequences were found to bind selectively to MCL-1, such as reverse BH3 motifs with helical conformation [67] and a variant of the BIM BH3 region, Bim_S_2A [68]. However, the proapoptotic properties of these peptides have not been characterized with the exception of Bim_S_2A which can induce BAK-dependent apoptosis when BCL-X_L_ is absent or neutralized [68].

#### Cross-linked NOXA-BH3 peptide

A short peptide derived from the BH3-only protein NOXA was biphenyl cross-linked in order to stabilize the helical conformation. The cross-linked BH3 peptide is capable of binding selectively to MCL-1 with an affinity comparable to that of NOXA (in the nM range) and it can induce death in a lymphoma cell line [69]. Conjugating this compound with ubiquitin further led to a MCL-1 antagonist with increased cell penetration and killing activity [70]. Whether the biphenyl-NOXA BH3 peptide actually induces BAX/BAK-dependent apoptosis is not known.

#### BIM SAHB and other stapled BH3 peptides

Another approach to stabilize the helical conformation has been to staple BH3 peptides. The first “stabilized α-helix of BCL-2 domain” (SAHB) was generated from the BH3 domain of BID [71]. A SAHB derived from the BH3 helix of BIM was recently described [72]. This BIM SAHB (BIM 21-mer, residues 146-166) binds with high affinity to BCL-X_L_, BCL-W, MCL-1 and A1, and it induces mitochondrial apoptosis in leukemia/lymphoma cell lines and tumor regression in an animal model of human leukemia [72]. Data indicate that the BIM SAHB displays the hallmarks of an authentic pan-BH3 mimetic. However, the cell permeability, binding affinity and biological activity of SAHB compounds are much debated issues [73, 74]. They seem to depend on the cell type inasmuch as BIM SAHB (residues 146-166) shows activity in hematologic but not fibroblastic cell lines [75].

Other relevant stapled peptides were described such as a SAHB modeled on the MCL-1 BH3 domain which was characterized as a selective MCL-1 inhibitor with proapoptotic properties in leukemia cell lines [76]. A SAHB derived from the BIM BH3 region was reported to be able to bind the MOMP effector BAX at a site that directly triggers its activation [77, 78]. These two compounds have further served as probes to identify small molecules with BH3 mimicry properties, respectively MIM-1 (see previous section Small organic molecules) and BAM-7 (described in next section Activators of proapoptotic BCL-2 proteins.).

#### BIM BH3-derived peptides of the XXA series

By combining computational design, combinatorial library screening and rational mutagenesis, a series of peptides derived from the BH3 region of BIM were engineered. The lead compound XXA1 binds to BCL-X_L_ with sub-nM affinity (and selectivity up to 1000-fold over other prosurvival proteins); it induces MOMP-dependent apoptosis in human platelets (for which BCL-X_L_ is a survival factor) and malignant cell lines [79]. Therefore, XXA1 may be considered as a new, putative BH3 mimetic highly specific for BCL-X_L_.

## ACTIVATORS OF PROAPOPTOTIC BCL-2 PROTEINS

Recent studies have used the BH3 mimetic concept to design small molecules having the ability to directly bind and activate proapoptotic members of the BCL-2 family, as described below.

### BAM-7

Walensky and colleagues first characterized a SAHB modeled on the BIM BH3 region and capable of binding the MOMP effector BAX at a site that directly triggers its activation [77, 78]. They then used a computational screen of small molecules for displacing their SAHB from its ligand BAX, to obtain a substituted pirazolone molecule called BAM-7; this compound can engage the BAX trigger site and induce BAX activation and apoptosis [80]. This was the first description of a small molecule capable of directly activating a proapoptotic BCL-2 family protein. The compound BAM-7 could be used to generate direct activators of BAX with high binding affinity.

### PUMA-BH3 peptide

Investigating the ability of the BH3-only proteins NOXA and PUMA to directly activate the MOMP effector BAK has highlighted a PUMA BH3 peptide that binds with nM affinity to the canonical binding pocket of BAK and induces its activation, leading to BAK-dependent MOMP and cell death [81, 82]. This study supports and extends others having indicated that different BH3 peptides as well as a BID SAHB can directly activate BAK [83-85]. These data therefore provide proof-of-concept that direct activators of BAK may be designed.

### PUMA SAHB

A SAHB derived from the PUMA-BH3 helix was found to display a dual activity: it can both engage all the prosurvival BCL-2 family proteins (and inhibit their activity) and directly bind and activate the proapoptotic BAX [86]. In addition, a cell permeable analog of this PUMA SAHB (capable of binding to BCL-2, MCL-1 and BAX) induces mitochondrial apoptosis in neuroblastoma cells [86]. These data demonstrate for the first time that PUMA BH3 is a dual prosurvival inhibitor and proapoptotic direct activator. It is therefore conceivable to design BH3 mimetics having such a dual capacity.

## CONCLUSIONS AND PERSPECTIVES

Since the emergence of the BH3 mimetic concept [4], targeting BCL-2 family proteins is considered to have a promising therapeutic potential especially for treating cancers [1, 2, 5]. Much progress has been made in this field of research with the identification of numerous inhibitors of prosurvival BCL-2 family proteins [6-16]. This field yet remains very challenging. The nature of the interaction between these compounds and their targets (protein/protein interaction, large and hydrophobic pockets) makes any program of drug discovery still difficult. Moreover, many putative BH3 mimetics were reported with minimal evidence of on-target effects. The low or modest binding affinity of some compounds is notably responsible for off-target effects. The difficulty to assess their mechanism of action can also be misleading [87].

Nevertheless, four small organic molecules targeting prosurvival BCL-2 family proteins have already entered clinical trials in cancer patients [15]. The clinical results with the two putative pan-BH3 mimetics obatoclax and AT-101 (that are now known to induce apoptosis mainly via off-target mechanisms) were disappointing. In contrast, the two authentic BH3 mimetics navitoclax and ABT-199 (which are both derived from the pioneer molecule ABT-737) proved to display anticancer efficacy particularly in hematologic malignancies: significant therapeutic effects were first observed with the BAD-like mimetic navitoclax and impressive results were further recorded with the BCL-2-specific ABT-199 that does not induce the thrombocytopenia exhibited by navitoclax (due to BCL-X_L_ inhibition). These data have unequivocally demonstrated that BH3 mimetic agents can expand the anticancer armamentarium in combination with conventional treatments.

None of the characterized BH3-derived peptides have so far progressed towards clinical evaluation. This does not mean that the approach to identifying BH3-derived peptides is less promising than the approach of screening small organic molecules. The former compounds indeed display liability and pharmacologic difficulties, whereas the latter are often prone to off-target effects. To achieve high affinity binding to the targets is a frequent issue for both types of compounds. A general conclusion favoring either approach cannot be drawn yet.

Updating the small molecules targeting BCL-2 family proteins (as recapitulated in Table 1) highlights not only new antagonists of prosurvival proteins but also the possibility to design novel types of BH3 mimetics capable of directly activating proapoptotic proteins.

**Table 1:** Main small molecules targeting BCL-2 family proteins and their characteristics

Compounds	Ref.	Targets	BH3 mimicry	Affinity	BAX/BAK apotosis	Antitumor in vivo	Clinical data/efficacy
***Antagonists of prosurvival proteins:***							
Obatoclax	17	BCL-2, BCL-XL, BCL-W, MCL-1, A1	Pan	μM	No	Yes	Yes/No
AT-101	22	BCL-2, BCL-XL, BCL-W, MCL-1, A1	Pan	sub-μM	Partly	Yes	Yes/No
Sabutoclax	24,26	BCL-2, BCL-XL, MCL-1, A1	Pan	sub-μM	Predominantly	Yes	
BIM SAHB	72	BCL-XL, BCL-W, MCL-1, A1	Pan	nM	Yes	Yes	
JY-1-106	58	BCL-XL, MCL-1	Pan	ND	Yes	Yes	
S1 derivative	54	BCL-2, BCL-XL, MCL-1	Pan	10-20 nM	Yes *	Yes *	
ABT-737	29	BCL-2, BCL-XL, BCL-W	BAD-like	nM	Yes	Yes	
Navitoclax	30	BCL-2, BCL-XL, BCL-W	BAD-like	nM	Yes	Yes	Yes/Yes
BM-1197	39	BCL-2, BCL-XL	BAD-like	sub-nM	Yes	Yes	
ABT-199	31,32	BCL-2	BCL-2-specific	sub-nM	Yes	Yes	Yes/Yes
WEHI-539	40	BCL-XL	BCL-XL-specific	nM	BAK-dependent		
A-1155463	41	BCL-XL	BCL-XL-specific	sub-nM	On-target mechanism	Yes	
XXA1	79	BCL-XL	BCL-XL-specific	sub-nM	MOMP-dependent		
Small molecule Mcl-1 inhibitor	46	MCL-1	NOXA-like	55 nM	Yes		
MIM-1	45	MCL-1	NOXA-like	μM	BAK-dependent		
TW-37	22,26	MCL-1	NOXA-like	sub-μM	Yes	Yes	
A-1210477	48	MCL-1	NOXA-like	sub-nM	On-target mechanism		
Biphenyl-NOXA BH3 peptide	69,70	MCL-1	NOXA-like	nM	ND		
***Direct activators of proapoptotic proteins:***							
BAM-7	80	BAX	BAX activator	ND	Yes		
PUMA BH3 peptide	82	BAK	BAK activator	nM	BAK-dependent		
***Dual prosurvival inhibitor and proapoptotic direct activator:***							
PUMA SAHB analog	86	BCL-2, MCL-1, BAX	Both pan-inhibitor and BAX activator	ND	Yes		

* Yes for the parental S1 compound but not determined for the S1 derivative; MOMP: mitochondrial outer membrane permeabilization; ND: not determined.

As regards the newly identified antagonists of prosurvival proteins, the first BH3 mimetic specific for BCL-X_L_ has been discovered with the highly potent small-molecule inhibitor A-1155463 displaying on-target, antitumor activity *in vivo.* The BH3 peptide derivative XXA1 also appears as a candidate of interest. The emergence of the compound A-1210477 - a potent MCL-1 inhibitor with a high binding affinity and an on-target mechanism of action - indicates that a NOXA-like BH3 mimetic specific for MCL-1 will rapidly be obtained. Other MCL-1 antagonists might likewise be probed such as the pioneer MIM-1, the remarkable Small Molecule Mcl-1 Inhibitor, and a biphenyl cross-linked NOXA peptide. The further characterization of TW-37 has revealed that modifications enhancing its affinity for MCL-1 could also lead to an authentic NOXA-like BH3 mimetic. The generation of pan-BH3 mimetics (which are needed for replacing obatoclax and AT-101) seems possible from the BIM SAHB or the gossypol derivative sabutoclax that are known to inhibit a broad range of prosurvival proteins. The small molecules S1-derivative and JY-1-106 were also described as starting points for designing pan-BH3 mimetics. Therefore, after the emergence of the BAD-like BH3 mimetic navitoclax and the BCL-2-specific ABT-199, the developments of NOXA-like, BCL-X_L_-specific and pan-BH3 mimetics will likely be achieved. Lastly, BM-1197 is added to navitoclax as another, potent BAD-like BH3 mimetic displaying a particular therapeutic potential for small cell lung cancer.

Moreover, a new dimension to targeting BCL-2 family proteins is provided by the discovery of two novel types of small molecules capable of directly activating proapoptotic members of the family. First, after the identification of the small molecule BAM-7 as a BAX activator, the finding that a PUMA BH3 peptide can activate BAK confirms the possibility to generate BH3 mimetics capable of directly activating the MOMP effectors. Second, the characterization of a PUMA SAHB analog which both activates BAX and antagonizes BCL-2 and MCL-1 indicates that it is possible to design BH3 mimetics having the dual activity of prosurvival inhibitor and proapoptotic activator.

Many efforts are obviously required to implement the clinical application of the novel types of BH3 mimetics. Nevertheless, the therapeutic targeting of BCL-2 family proteins already appears as a potent weapon for overcoming the apoptotic resistance in cancers and improving the treatments of patients.
